# Novel Lectins from *Bauhinia* with Differential *N-*Glycan Binding Profiles

**DOI:** 10.1021/acsomega.5c00961

**Published:** 2025-04-08

**Authors:** Vinicius J. S. Osterne, Lara D. Lima, Messias V. Oliveira, Vanir R. Pinto-Junior, Cornevile C. Neto, Sarah E. G. Correia, Clara Suarez, Els J. M. Van Damme, Sonia Serna, Niels C. Reichardt, Kyria S. Nascimento, Benildo S. Cavada

**Affiliations:** † Department of Biochemistry and Molecular Biology, BioMol-Lab, Federal University of Ceara, Fortaleza 60020-181, CE, Brazil; ‡ Laboratory of Biochemistry and Glycobiology, Department of Biotechnology, Ghent University, 9000 Ghent, Belgium; § CIC biomaGUNE, Basque Research and Technology Alliance (BRTA), 20014 Donostia-San Sebastián, Spain; ∥ Biomedical Research Networking Center in Bioengineering, Biomaterials, and Nanomedicine (CIBER-BBN), 20014 Donostia-San Sebastián, Spain

## Abstract

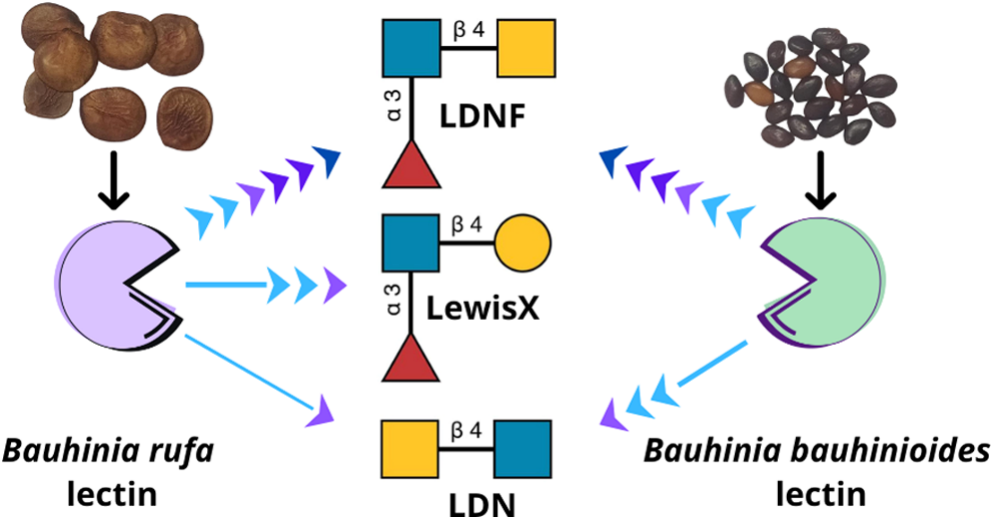

The specific interaction of lectins with carbohydrates and glycoconjugates grants these proteins a distinct ability to decode the glycocode. Essential for various biological processes in all organisms, this carbohydrate-binding activity also establishes lectins as valuable tools in fields such as glycomics, medicine, and biotechnology. Considering that the discovery of novel lectins with unique binding profiles is particularly relevant, this study investigated the binding specificity of two lectins extracted from *Bauhinia* seeds toward simple sugars, *N*-glycans and *O*-glycans. The combination of agglutination-inhibition assays and glycan arrays revealed subtle differences in the binding of the lectins to galactosides and glycans containing specific motifs, such as LewisX, LacdiNAc, and fucosylated LacdiNAc. Despite slight differences in carbohydrate-binding patterns, both lectins showed similar results in toxicity assays using *Artemia salina* nauplii and cytotoxicity assays on cancer cell lines, with neither lectin exhibiting significant toxicity. Additionally, both lectins demonstrated low cytotoxicity toward HeLa (cervical adenocarcinoma), HT1080 (fibrosarcoma), and NHDF (normal fibroblasts), even at concentrations up to 125 μg/mL. Analysis of the partial amino acid sequences of these lectins revealed conserved residues compared to other lectins of the genus, as well as secondary structure conformations similar to those of other legume lectins. This research represents a significant advancement in the understanding of lectins from the genus *Bauhinia*, and future structural studies could further elucidate the interactions of these proteins with their ligands, providing fundamental insights into their biological functions and paving the way toward potential applications.

## 1 Introduction

Lectins are a diverse and widespread group of proteins found in all kingdoms of life. They are capable of binding specifically and reversibly to carbohydrate structures. Lectins are of non-immune origin and should not be confused with carbohydrate-specific immunoglobulins. Unlike enzymes that act on carbohydrates, lectins bind without modifyng the carbohydrate ligand.^1^ The ability of lectins to recognize carbohydrates is essential for various biological functions, as carbohydrates serve as mediators in cellular communication and processes such as differentiation, growth, proliferation, defense, and inflammation.^2,3^ As one of the main groups of carbohydrate-binding proteins, lectins interact with cell-surface glycans, making them powerful biological tools with applications across numerous fields. These include biomedical diagnostics,^4^ drug delivery systems,^5^ the development of antitumor and antiviral drugs,^6^ among others.^7^ Within the lectin field, plant lectins, particularly those extracted from plants of the *Leguminosae* family, have been studied extensively. This legume family is traditionally divided into three subfamilies: Papilionoideae, Caesalpinioideae, and Mimosoideae. It is widely accepted that lectins characterized from species belonging to the same family present a high degree of structural similarity. However, they can vary significantly in their biological activities, carbohydrate specificities, and biochemical properties.^8^

Being the most researched lectin family, considerable information is available regarding the biochemical properties and carbohydrate-binding specificity of legume lectins. While these lectins typically bind mono- and oligosaccharides, it is unlikely that these proteins bind these molecules in a biological context, simply because the monosaccharides are not readily available in cells. Researchers revealed that these lectins can also recognize complex carbohydrates and glycoproteins, generally with higher binding affinities for di-, tri-, and tetrasaccharides compared to monosaccharides.^9^ Based on carbohydrate inhibition assays, legume lectins are classified into five groups: mannose/glucose-specific, *N*-acetyl-d-galactosamine/galactose-specific, *N*-acetyl-d-glucosamine-specific, l-fucose-specific, and α-2,3 sialic acid-specific lectins.^10^ The specific interaction with glycoconjugates has facilitated purification processes using affinity matrices with immobilized carbohydrates, glycans, or glycoproteins. Recent advances, including glycan microarrays, confirm and expand on prior findings and provide insights into the specificity of lectins toward glycan structures.^11^

Although *Bauhinia* lectins were studied as early as 1978.^12,13^ However, despite the genus comprising around 300 catalogued species,^14,15^ only ten lectins from seven species: *Bauhinia bauhinioides*, *Bauhinia forficata*, *Bauhinia monandra*, *Bauhinia pentandra*, *Bauhinia purpurea*, *Bauhinia ungulata*, *Bauhinia variegata* and *Bauhinia catingae*—have been identified to date.^16−19^ These lectins have been extracted from various plant parts (leaves, seeds, roots) and purified using classical protein chemistry techniques, such as aqueous extraction and ammonium sulfate fractionation in combination with chromatographic methods, including affinity, ion exchange, and molecular exclusion chromatographies. The protocols were highly successful in isolating and purifying *Bauhinia* lectins, facilitating detailed biochemical characterization.^17^

The biological activities of *Bauhinia* lectins are remarkably diverse, encompassing anti- and pro-inflammatory, anticoagulant, anticancer, insecticidal, antinociceptive, antifungal, immunomodulatory, antiviral, antibacterial, and antibiofilm effects.^17^ This wide range of activities makes them promising candidates for biotechnological applications. Structurally, the sparse data on *Bauhinia* lectins reveal a typical legume-lectin fold with a metal-binding site and a carbohydrate-recognition domain.^20−22^ All *Bauhinia* lectins predominantly bind to galactosides, such as galactose, lactose, N-acetylgalactosamine, and melibiose (Galα-1,6Glc), with the highest affinity for *N*-acetylgalactosamine.^23,24^ Only a small subset of the lectins interact with glucose, glucuronic acid, and raffinose.^13^

Despite the considerable progress in understanding these lectins, further research is needed to (1) identify new lectins from *Bauhinia* species and elucidate their primary and three-dimensional structures, (2) provide additional data on their binding specificity beyond mono- and disaccharides, and (3) investigate biological effects of these lectins. In this study, the carbohydrate-binding specificities of two *Bauhinia* lectins—*Bauhinia rufa* (BRL) and *B. bauhinioides* (BBL)—were examined using glycan microarrays. Interactions with a range of glycans were assessed, including *N*-glycans (high mannose and complex types), terminal epitopes, fucosylated cores, and *O*-glycan cores. The toxicity of both lectins toward *Artemia* nauplii and their cytotoxicity against two cancer cell lines were also examined. Together, these analyses provide valuable insights into the biochemical properties and potential applications of *Bauhinia* lectins.

## 2 Materials and Methods

### 2.1 Lectin Purification

The lectin from *B. rufa* (BRL) was obtained from seed flour. Soluble proteins were extracted in 100 mM Tris-HCl buffer, pH 7.6, containing 150 mM NaCl (1:10 (g/mL)), for 4 h. The extract was centrifuged at 9000*g* for 30 min at 4 °C, and proteins in the supernatant were precipitated with ammonium sulfate added to 60% saturation, resuspended in the same extraction buffer, and applied to an agarose-lactose affinity matrix. Unbound proteins were eluted with extraction buffer, and the lectin was eluted with 100 mM lactose in the same buffer. The lectin from *B. bauhinioides* (BBL) was purified following the protocol of Silva and colleagues (2011).^16^

All protein fractions were analyzed by spectrophotometry at 280 nm. The purity and yield of lectins were quantified by the Bradford assay.^25^ To monitor the activity of the lectins, hemagglutination assays were performed using rabbit and human erythrocytes. Hemagglutination titers were measured in terms of hemagglutinating units (HU) as the inverse of the highest dilution capable of showing visible hemagglutination. In the sugar-inhibition assays, the following carbohydrates were used: d-glucose, d-mannose, α-methyl-d-glucopyranoside, α-methyl-d-mannoside, d-galactose, α-lactose, *N*-acetyl-d-glucosamine, *N*-acetyl-d-galactosamine, l-rhamnose, l-fucose, melibiose, and mucin glycoprotein.

### 2.2 Glycan Arrays

Glycan arrays were used to assess the carbohydrate-binding specificity of BRL and BBL (see Supporting Figure S1). These synthetic microarrays contained 142 glycan structures, prepared as previously established.^26,27^ BRL and BBL were fluorescently labeled using Alexa Fluor 555 succinimidyl ester (Fisher Scientific) according to the manufacturer’s guidelines. Excess dye was removed with an Amicon 10 kDa filter. Labeled lectins were diluted in a 25 mM Tris-HCl buffer containing 150 mM NaCl, pH 7.5, supplemented with 2 mM CaCl_2_, 2 mM MgCl_2_ and 0.01% Tween 20. Lectin solutions, adjusted to an absorbance (A280 nm) of 0.2, were incubated on the glycan microarray in the dark at room temperature for 1 h to allow binding to take place. Following incubation, the arrays were washed thrice with the same buffer and water to remove unbound material. The microarrays were dried using a slide spinner and fluorescence was analyzed using an Agilent G265BA microarray scanner (Agilent, Santa Clara, CA). Fluorescence was expressed as relative fluorescence units (RFU) and presented as histograms showing the mean ± SD from four replicate spots (after subtracting local background). The results were visualized as relative fluorescence units, where higher fluorescence intensities reflected stronger lectin-glycan interactions.

### 2.3 Mass Spectrometry Analysis

The partial amino acid sequence of BRL was determined using mass spectrometry. A 4.5 μL aliquot of peptides, obtained through digestion of the lectin with trypsin, chymotrypsin, pepsin, and Glu-C, was separated using a C18 (100 μm × 100 mm) RP-nanoUPLC system (nanoAcquity, Waters, Milford, MA) coupled to a Q-Tof Premier mass spectrometer (Waters, Milford, MA). The separation was carried out at a flow rate of 0.600 μL/min with a gradient of 2 to 90% acetonitrile in 0.1% formic acid over 20 min. The source voltage (nanoelectrospray), cone voltage, and temperature were set at 3.5 kV, 30 V, and 80 °C, respectively. The three most intense MS peaks detected were selected for fragmentation to generate MS/MS data. For analysis of endogenous cleavage peptides, real-time deletion was employed. MS/MS spectra were processed using MassLynx 4.1 software, and the resulting peptide sequences were analyzed via BLASTp.^28^ The identified peptides were further compared using the Blastp database,^29^ and aligned with similar lectins using ClustalOmega^30^ and ESPript 3 tools.^31^

### 2.4 *Artemia* Nauplii Assays

The toxicity of BRL and BBL lectins was tested against *Artemia salina* nauplii following the methodology described by Arruda et al. and Cavada et al.^32,33^ using 24-well Linbro plates. A 2-fold serial dilutions of the lectins were prepared in sterile seawater, with concentrations ranging from 250 to 31.25 μg/mL. Ten nauplii were introduced into each well and incubated for up to 48 h. Viability was assessed at 24 and 48 h by counting live nauplii, identified by their motility. Two controls were included: a seawater-only control and a lectin + carbohydrate control, the latter to evaluate the importance of the carbohydrate-binding activity for lectin toxicity. The lectin from *Canavalia brasiliensis* (ConBr) was used as a positive control. Each experimental condition was performed in triplicate, and the data obtained were analyzed to evaluate lectin toxicity. Mean values and standard deviations of live nauplii counts were compared to control groups. The sugars applied for inhibition of the *Bauhinia* lectins and ConBr were GalNAc and methyl-α-d-mannopyranoside, respectively, both at 100 mM concentration.

### 2.5 Cytotoxicity Assays

HeLa (ATCC CCL-2), HT-1080 (ATCC CCL-121) and NHDF (PCS-201-012) cells were obtained from the American Type Culture Collection (ATCC, Manassas, VA). Cells were cultured in Dulbecco’s Modified Eagle Medium, supplemented with 10% (v/v) fetal bovine serum and 1% (v/v) penicillin/streptomycin (all from ThermoFisher Scientific, Waltham, MA). Cultures were maintained in T75 flasks at 37 °C in a humidified atmosphere containing 5% CO_2_. Subculturing was performed approximately twice per week when cells reached 80% confluency, with splitting ratios optimized to account for the doubling times of each cell line.

Cell viability was assessed in 96-well plates following exposure to BRL and BBL. A 2-fold serial dilution of each lectin in medium was prepared to achieve final concentrations of 125, 62.5, 31.2, 15.6, and 7.8 μg/mL. Cells were counted with a hemocytometer and seeded at 5000 cells/well in a final volume of 100 μL. Cells were incubated at 37 °C with 5% CO_2_ for 24 or 48 h. After incubation, the medium was removed, and 100 μL of a 1× solution of PrestoBlue HS (ThermoFisher, Waltham, MA) in phosphate-buffered saline (PBS) was added. The plates were incubated for 1 h at 37 °C in the dark, followed by fluorescence measurement using an Infinite F200 Pro plate reader (Tecan, Männedorf, Switzerland), with filters set to 560 nm excitation and 590 nm emission. Control wells containing untreated cells were included to determine baseline viability, along with negative control wells containing only 100 μL of a 1× PrestoBlue HS solution in PBS to evaluate background signal. Each experiment was performed twice independently, with four technical replicates per condition. Data were analyzed using two-way ANOVA followed by Dunnet post-hoc test. Statistical significance was considered at p < 0.05.

## 3 Results and Discussion

### 3.1 Galactosides Make the Basis of the Carbohydrate-Specificity in BRL and BBL

Initial hemagglutination assays were performed using human erythrocytes from blood types A, B, and O; however, no agglutination activity was observed. Subsequently, assays were conducted using rabbit erythrocytes, and BRL exhibited robust agglutination activity with these cells. Agglutination-inhibition assays revealed that both lectins exhibit specificity toward galactosides. BRL showed strong inhibition by, from the highest to lowest affinity, α-lactose, d-galactose, *N*-acetyl-d-galactosamine (GalNAc), melibiose, and rhamnose. Similarly, BBL was inhibited by α-methyl-d-galactopyranoside, GalNAc, D-galactose, melibiose, and l-rhamnose, consistent with previous data on *Bauhinia* lectins.^34^ Neither lectin showed was inhibited by mannosides at concentrations up to 100 mM (Table 1). Notably, the lectins did not induce agglutination of human erythrocytes. While rabbit erythrocytes are known to present a variety of glycans on their surface, including sialylated structures and polylactosamine extensions, their complete glycan composition, and particularly its correlation with blood types, has not yet been fully characterized.^35^ Theoretically, this incomplete characterization, combined with BRL’s galactoside preference, might suggest rabbit erythrocytes are a suboptimal choice for these assays. However, the strong agglutination response indicates that suitable glycan targets for BRL are indeed present on the rabbit erythrocyte surface.

**Table 1:** Specificity of BRL and BBL for Different Carbohydrates Using Hemagglutination-Inhibition Assays^a^

	minimum inhibitory concentration (mM)	
carbohydrates	BRL	BBL
d-glucose	-	-
d-mannose	-	-
d-galactose	1.56	3.16
l-rhamnose	25	25
l-fucose	-	-
l-fructose	-	-
d-arabinose	-	-
α-methyl-d-glucopyranoside	-	-
α-methyl-d-mannoside	-	-
α-methyl-d-galactopyranoside	-	1.56
*N*-acetyl-d-glucosamine	-	-
*N*-acetyl-d-galactosamine	0.78	1.56
sucrose	-	-
α-lactose	6.25	3.16
melibiose	6.25	6.25

a (−) Absence of hemagglutination inhibition at concentrations up to 100 mM.

*Bauhinia* lectins are generally known to recognized-galactose, GalNAc, and in some cases d-glucose. Structural studies of *B. ungulata* lectin (BUL) suggest that the preference for GalNAc over d-galactose arises from the *N*-acetamide group substituent at C-2, which enables additional interactions within the carbohydrate-recognition domain.^23,24^ This preferential binding to GalNAc has also been observed in other galactoside-binding lectins, such as soybean agglutinin,^36^*Luetzelburgia auriculata* lectin,^37^ and *Moluccella laevis* lectin.^38^ Consistent with the specificity toward galactosides, lactose binding has been documented in several *Bauhinia* lectins, including those from *B. variegata* var. candida,^17^*B. pentandra*,^16^*B. ungulata*,^23^*B. variegata*,^39^*B. purpurea*,^40^ with minimum inhibitory concentrations (MIC) of 0.25, 0.557, 0.96, 1.15 and 1.6 mM, respectively. Additionally, lectins such as BPL (from *B. pentandra*), BPH (from *B. purpurea*), and BvL (from *B. variegata*) demonstrated specificity for melibiose, with MICs of 0.443, 1.6, and 7.8 mM, respectively. Lactose and melibiose share a significant structural relationship, both being disaccharides composed of galactose and glucose. However, their differing glycosidic linkages—α-1,6- for melibiose and β-1,4-for lactose—impact their binding interactions.^41^

BRL also exhibits specificity towards rhamnose, a sugar whose recognition by lectins may may contribute to host defense mechanisms. For instance, lectins from the coral *Pocillopora damicornis* and the sea bass *Dicentrarchus labrax* bind rhamnose, facilitating bacterial agglutination through the recognition of surface lipopolysaccharides and lipoteichoic acids.^42−44^ Since the rhamnose biosynthesis pathway is absent in humans, targeting this sugar presents a potential therapeutic approach for bacterial infections.^45^

### 3.2 BRL and BBL Exhibit Distinct Preferences for Fucosylated Galactosylated *N*-Glycans

**Figure 1:**
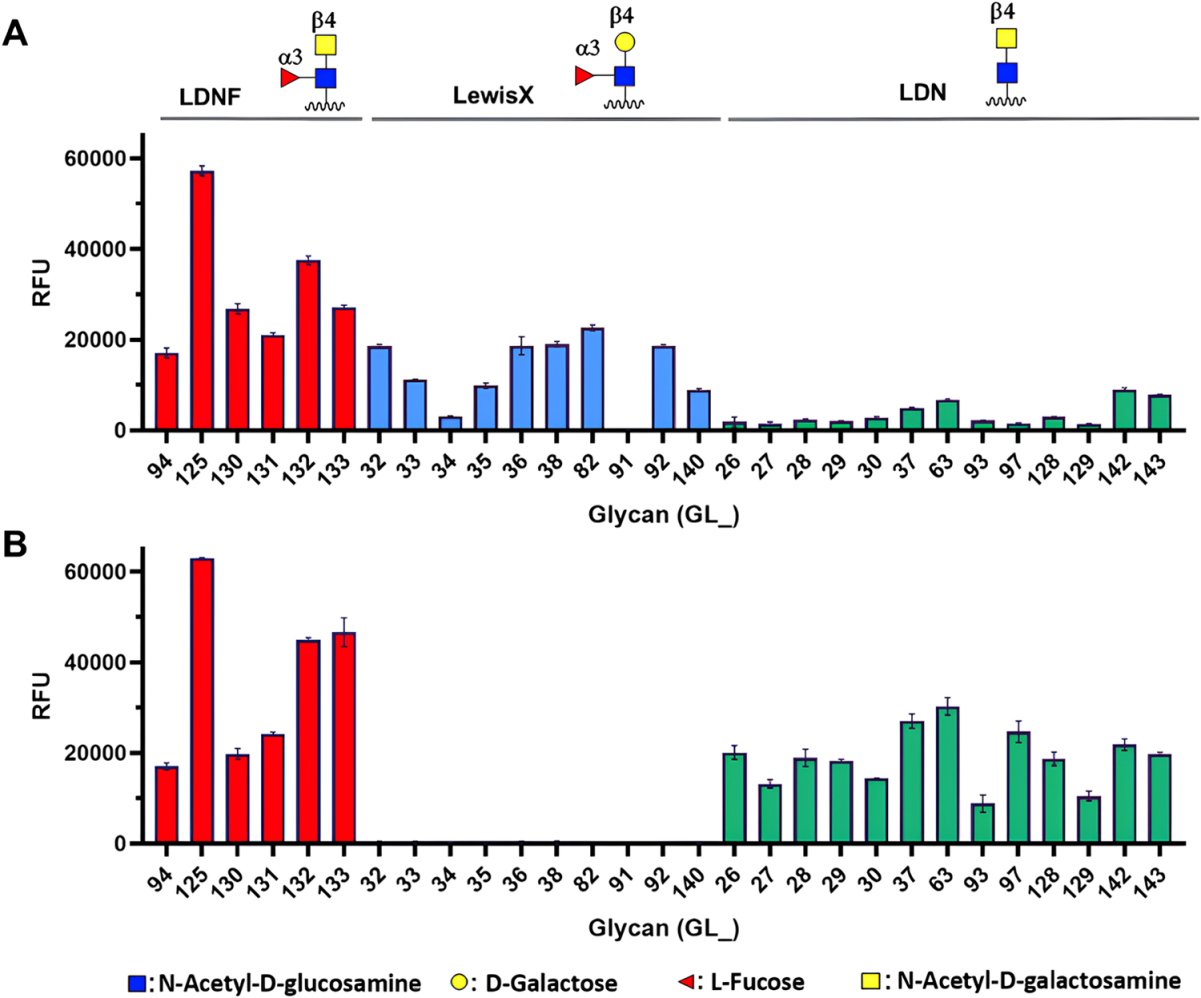
Glycan array screening of BRL (A) and BBL (B). Each histogram represents the mean RFU (relative fluorescence unit) values for four spots with SD (standard deviation) of the mean. Only select glycan structures are represented and grouped considering their terminal epitopes: LDNF (red bars), Lewis X (blue bars) and LDN (green bars). Glycan structures included in the microarray are available in Figure S1 and full data from microarray experiments in Figure S2.

The carbohydrate-binding properties of BRL and BBL were analyzed using a glycan microarray featuring a diverse range of chemo-enzymatically prepared carbohydrates with a strong focus on *N*-glycans, including mammalian, plant and invertebrate glycans.^26,46^ Positive interactions with fluorescently labeled BRL and BBL were assessed by measuring relative fluorescence units with a microarray scanner. Results are graphed as histograms, where higher fluorescence indicates greater binding (Figure 1, only selected glycans were represented. See SI for glycan structures included in the microarray and full microarray data). The results revealed that BRL interacts with complex *N*-glycans containing three different motifs: Galβ1–4(Fucα1–3)GlcNAc (Lewis X or LeX), GalNAcβ1–4GlcNAc (LacdiNAc or LDN), and GalNAcβ1–4(Fucα1–3)GlcNAc (α1,3-fucosylated LDN or LDNF) motifs (e.g., GL82, GL63, GL125). BRL showed a preference for LDNF motifs (GL130, GL131) and the biantennary N-glycan in which both branches contained LDNF motifs (GL125) showed the highest interaction among all structures tested in the microarray. BRL also interacted with glycans featuring LeX motifs including both biantennary (GL82 and GL92) and monoantennary N-glycans (GL35). Core modifications, including β-1,2-xylose (GL36) and core fucosylation (GL38) did not significantly affect binding. Finally, BRL showed weak binding to *N*-glycans with terminal LDN motifs (GL63) compared to those with LDNF or LeX motifs, emphasizing the importance of terminal fucosylation in the interaction in its glycan recognition.

BBL preferentially recognized glycans displaying LDNF and LDN motifs. As previously discussed for BRL, BBL also demonstrated binding to complex *N*-glycans containing LDNF motifs, including biantennary glycans with LDNF on one branch (GL130, GL131) and showed greated binding to glycans carrying LDNF on both branches (GL125). However, unlike BRL, BBL did not interact with biantennary *N*-glycans containing LeX motifs (e.g., GL92). Unlike BRL, BBL bound effectively to *N*-glycans with terminal LDN motifs (GL63). Modification of the *N*-glycan core by β-1,2-xylose and core fucosylation (GL29, GL37) did not impede binding.

Among *Bauhinia* genus lectins, BfL was evaluated for carbohydrate-binding specificity using a glyco-antigen microarray of approximately 500 components. BfL selectively bound glycans with GalNAc residues at the nonreducing termini, with the strongest binding observed for the Tn-antigen, a tumor-associated carbohydrate composed of GalNAc α-linked to serine or threonine residues in a polypeptide chain. Modifications to GalNAc often eliminated BfL binding, and no interactions were observed with glycopeptides containing GlcNAc-α, Fuc-α, Man-α, or Gal-α residues.^20^ Further studies have shown that lectins such as BPL can serve as predictors of cancer recurrence and tumor characteristics.^47^ Microarray analyses revealed that BPL binding patterns could distinguish between normal and cancerous tissues, including gastric and endometrial cancers, and correlated with histological type and mutation status. Additionally, glycan profiling of ovarian cancer cells demonstrated that specific lectins, including BPL, could distinguish between drug-sensitive and drug-resistant cell lines, potentially aiding in predicting chemotherapy outcomes.

### 3.3 Conserved Carbohydrate-Binding and Metal Ion Coordination Regions Identified in BRL and BBL Sequences

Partial amino acid sequences of both lectins have been obtained, revealing conserved regions similar to other *Bauhinia* lectins. These regions primarily consist of residues involved in carbohydrate binding and metal ion coordination, such as Ca^2+^ and Mn^2+^, as seen in structural models of *B. forficata* (PDB id: 5T52) and *Griffonia simplicifolia* (PDB id: 1LEC). For BRL, 159 amino acid residues were de novo sequenced using MassLynx 4.1 and the MaxEnt1 algorithm. BLASTp analysis of these peptides showed identity percentages of 75.8, 74.5, 70.5, 61.4, 59.6, and 51.5% compared to lectins from *B. variegata*, *B. purpurea*, *B. ungulata*, *B. forficata* (isoform 1), *G. simplicifolia*, and *B. forficata* (variant 2), respectively.

**Figure 2:**
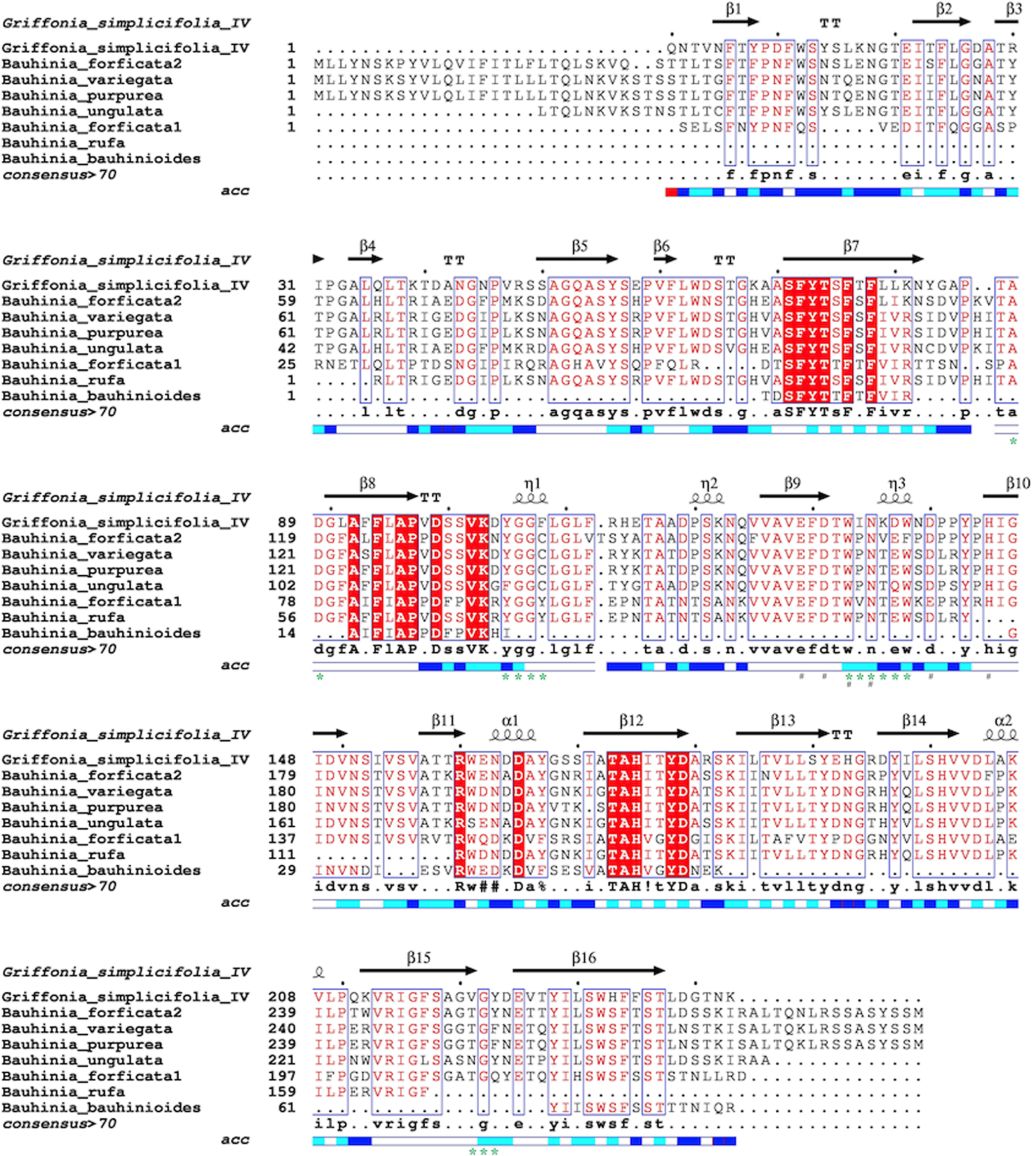
Sequence alignment of BRL and BBL with other Cercidoideae subfamily lectins. Residues that constitute the carbohydrate recognition domain (*) and metal-binding site (#). The relative accessibility of each residue is rendered as blue-colored boxes shown in the last line of each block. Higher intensity of blue reflects better accessibility.

For the BBL lectin, BLASTp analysis of 76 previously published, though unaligned, amino acid residues revealed identity percentages of 35.26, 48.94, 44.68, 27.12, and 27.97% with lectins from *B. forficata* (variant 2), *B. forficata* (variant 1), *B. variegata*, *B. ungulata*, and *B. purpurea*, respectively. Both BRL and BBL also demonstrated significant sequence similarity with lectin IV from *G. simplicifolia*, a galactose-specific lectin. Multiple sequence alignment revealed conserved regions in the BRL sequence, particularly in regions involved in carbohydrate and metal ions binding. However, due to the limited availability of sequenced peptides such regions were not observed in the partial protein sequence of BBL (Figure 2).

In reviews by Cavada et al. and Cagliari et al., multiple alignments of lectins from various *Bauhinia* species and other Cercidoideae plants showed that the primary structure of most *Bauhinia* lectins has not been fully characterized. These lectins exhibit a high degree of similarity, with sequence identity ranging from 80 to 89% for fully sequenced lectins such as BFL, BPA, BUL, and BVL and their isoforms obtained respectively from *B. forficata*, *B. purpurea*, *B. ungulata*, and *B. variegata*. Comparisons with other members of the Cercidoideae subfamily, including *G. simplicifolia* lectin, revealed over 70% similarity, while more distantly related lectins, such as ConA, showed approximately 40% sequence similarity. The strong sequence conservation supports the use of lectins as chemotaxonomic markers at the family and subfamily levels.^17,48^

Using the ESPript 3.0 program, secondary structures of BRL and BBL were analyzed based on available sequences. Alignments with other *Bauhinia* lectins and *G. simplicifolia* lectin IV (1LEC), which shares the highest primary structure similarity, revealed predominance of β-sheets with some α-helices. Secondary structure studies of other Bauhinia lectins, including *B. forficata*, *B. purpurea*, *B. ungulata*, and *B. variegata*, showed similar patterns. Circular dichroism analysis further confirmed a high content of β-sheet but low proportion of α-helix, characteristic for legume lectin structures.^48^

### 3.4 BRL and BBL are Nontoxic to *A. salina*

The lethality test using *A. salina* has been regarded as a preliminary assay for evaluating compounds with potential applications in cancer detection, antiviral activity, and insecticide development, highlighting their cellular toxicity relevance.^49^ After 24 h, the viability of nauplii exposed to BRL and BBL remained above 80%, similar to the control groups. In contrast, ConBr exhibited an average nauplii viability of 60% at a concentration of 250 μg/mL, while no significant reductions were observed at other concentrations. At 48 h, no significant differences were observed in the viability of nauplii across all tested concentrations compared to controls. In contrast, BBL caused nearly a 50% reduction in viability of nauplii at 250 and 125 μg/mL, while ConBr also decreased viability by 50%. Notably, the toxicity of ConBr and BBL was dependent on their carbohydrate-recognition domains, as blocking with specific carbohydrates reduced their toxicity. The slight decline in nauplii viability in the controls over time was anticipated due to the absence of food. The subtle differences in toxicity between BRL and BBL, particularly at concentrations of 250, 125, and 62.5 μg/mL, may be attributed to their varying affinities for galactosides (Table 1) and glycans (Figure 1). A notable discrepancy in toxicity between these lectins and ConBr could be attributed to the carbohydrates composition of *A. salina* nauplii. It is likely that more mannosides are more abundant than galactosides. In fact, glycomic mapping of invertebrates has identified at least 11 carbohydrate types, including glucose, mannose, galactose, GlcNAc, GalNAc, xylose, fucose, NeuAc, NeuGc, GlcA, and iduronic acid, with a prevalence of *N*-glycans terminating in mannosides.^50^

This study demonstrated that BRL and BBL did not present significant toxicity towards *A. salina* nauplii at the concentrations tested, classifying them as nontoxic in this model. These findings indicate their potential for use in cell culture and assays requiring nontoxic proteins or molecules. Few studies have examined the toxicity of *Bauhinia* lectins; for instance, *B. monandra* leaf lectin exhibited low toxicity, not affecting nauplii survival at concentrations between 250 and 1000 μg/mL.^51^

### 3.5 BRL and BBL Exhibit Minimal Cytotoxicity across Tested Cell Lines

Viability assays were performed to assess the effects of BRL and BBL on NHDF, HT1080, and HeLa cells at final concentrations of 125, 62.5, 31.2, 15.6, and 7.8 μg/mL. The results showed that both BRL and BBL displayed minimal cytotoxicity across all tested cell lines. Even at the highest concentration tested (125 μg/mL), viability was only slightly reduced. After 24 and 48 h of incubation, viability remained above 75% in most cases and dose-dependent effects were marginal, with no significant differences observed between the two lectins.

These results stand in stark contrast to other legume lectins, most of which demonstrate significant antiproliferative effects by activating programmed cell death pathways such as apoptosis, autophagy or necrosis, depending on the lectin and cell linein cancer cells. These effects are are thought to result from the interaction of lectins with specific glycosylated targets.^52^ A number of *Bauhinia* lectins have also demonstrated remarkable antiproliferative potential. For instance, *B. forficata* lectin (BfL) has been shown to negatively impact the viability of MCF-7 human breast cancer cells by inducing necrosis, DNA fragmentation, and cell cycle arrest, while also inhibiting cell adhesion and activating apoptosis pathways. These effects are closely tied to the lectin’s ability to modulate integrin subunit expression, disrupt cell-matrix interactions, and activate mitochondrial-dependent cell death mechanisms.^53^

Similarly, the lectin from *B. ungulata* (BUL) exhibited a dose-dependent antiproliferative effect against HT-29 human colon adenocarcinoma cells, an activity attributed to its carbohydrate-binding specificity, particularly for glycoproteins on cancer cell surfaces. This binding may disrupt critical signaling pathways necessary for tumor growth and survival.^18^ Furthermore, a second lectin from *B. forficata*, referred to as BfL-II, displayed potent antiproliferative activity against both MCF-7 and HT-29 cancer cell lines, resulting in over 80% inhibition at higher lectin concentrations.^54^

## Conclusions

This study evaluated the carbohydrate-binding specificity and biological activities of two lectins, BRL and BBL, isolated from *B. rufa* and *B. bauhinioides* seeds. Using agglutination-inhibition assays and glycan microarrays, we demonstrated that these lectins predominantly bind galactosides and exhibit distinct affinities for glycans with motifs such as LewisX, LacdiNAc, and fucosylated LacdiNAc (LDNF). Despite differences in binding profiles, both lectins exhibited similarly low toxicity in *A. salina* and human cell line assays, including cancerous (HeLa and HT1080) and normal fibroblasts (NHDF). Partial sequencing and structural analysis revealed a remarkable similarity between these lectins and other *Bauhinia* lectins, with conserved residues in the binding sites. Their glycan-binding specificity may underlie the limited toxicity to *Artemia* nauplii and weak cytotoxic effects in cancer cells compared to other legume lectins. While plant lectins are mainly recognized for their role in plant defense and development, they also exhibit diverse biological activities with biotechnological relevance. This study characterized the properties and biological activities of BRL and BBL lectins outside the plant organism, including their effects on cell viability. Furthermore, the specificity data of these lectins suggest potential for broader applications to be explored in the future. Overall, our work provides insights into the glycan-binding speificities and biological activity profiles of *Bauhinia* lectin, offering a starting point for future research on their applications in biotechnology, medicine, and glycomics.

## Data Availability

The data underlying this study are not publicly available as no suitable public repository exists for the data set types involved. The data are available from the corresponding author upon reasonable request. No registration or data use agreement is required for access.

## Supporting Information Available

Images related to the glycan array analysis of Bauhinia seed lectins, including the tested glycans, glycan structures and histograms containing relative fluorescence units (PDF)
