# Supplementary material for: Novel Lectins from Bauhinia with Differential N-Glycan Binding Profiles
Source: ACS Omega. 2025 Apr 8;10(15):15637–45. doi: 10.1021/acsomega.5c00961 (PMC12019499; doi:10.1021/acsomega.5c00961)
Supplement: Supplementary file 1 — ao5c00961_si_001.pdf [file ao5c00961_si_001.pdf]

Electronic Supplementary Information

Novel lectins from *Bauhinia* with differential *N*-glycan binding profiles

Vinicius J. S. Osterne[1,2], Lara D. Lima[1], Messias V. Oliveira[1], Vanir R. Pinto-Junior[1], Cornevile C. Neto[1], Sarah E. G. Correia[1], Clara Suarez[1], Els J. M. Van Damme[2], Sonia Serna[3], Niels C. Reichardt[3,4], Kyria S. Nascimento[1,*] and Benildo S. Cavada[1,*]

[1]Department of Biochemistry and Molecular Biology, BioMol-Lab, Federal University of Ceara, Fortaleza 60020-181, CE, Brazil
[2]Laboratory of Biochemistry and Glycobiology, Department of Biotechnology, Ghent University, 9000 Ghent, Belgium
[3]CIC biomaGUNE, Basque Research and Technology Alliance (BRTA), 20014 Donostia-San Sebastián, Spain.
[4]Biomedical Research Networking Center in Bioengineering, Biomaterials, and Nanomedicine (CIBER-BBN), 20014 Donostia-San Sebastián, Spain.
*Corresponding authors: K.S.N. (kyriasantiago@ufc.br); B.S.C. (bscavada@ufc.br)

Figure S1. A) Pictogram representation of glycan structures included on the microarray. B) Specification of glycosidic bond configurations.

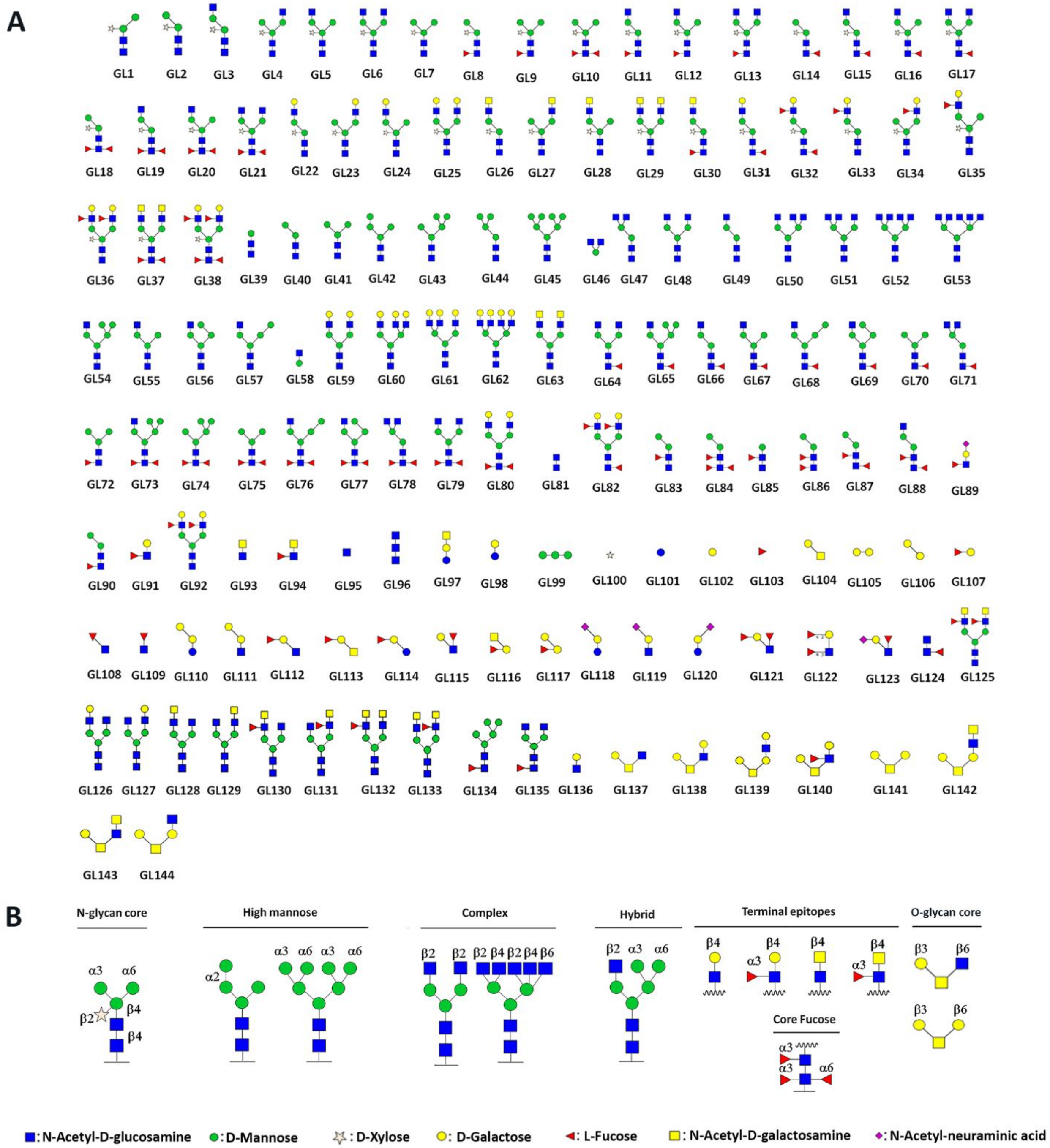

Figure S2. Microarray screening of BRL (A) and BBL (B). Each histogram represents the mean RFU (relative fluorescence unit) values for four spots with SD (standard deviation) of the mean.

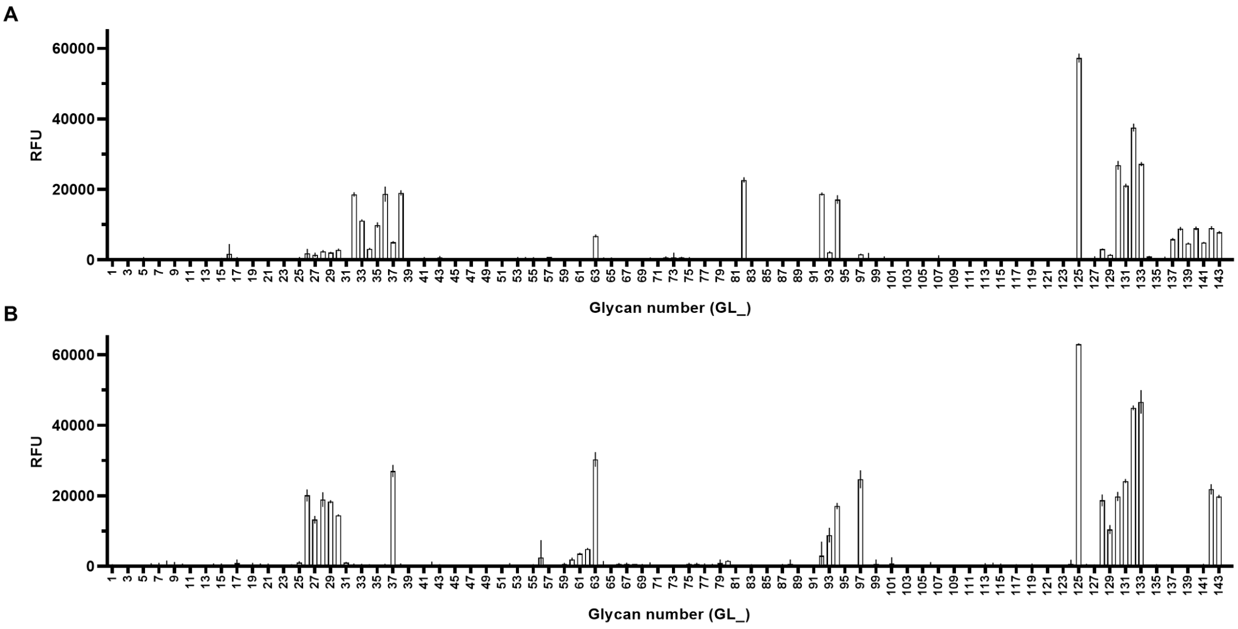
A
RFU
60000
40000
20000
0
Glycan number (GL_)
B
RFU
60000
40000
20000
0
Glycan number (GL_)
